# Plant vacuole morphology and vacuolar trafficking

**DOI:** 10.3389/fpls.2014.00476

**Published:** 2014-09-24

**Authors:** Chunhua Zhang, Glenn R. Hicks, Natasha V. Raikhel

**Affiliations:** Center for Plant Cell Biology and Department of Botany and Plant Sciences, University of California at RiversideRiverside, CA, USA

**Keywords:** vacuole morphology, vacuole fusion, Rab GTPases, SNAREs, HOPS complex, lipids, actin cytoskeleton, chemical biology

## Abstract

Plant vacuoles are essential organelles for plant growth and development, and have multiple functions. Vacuoles are highly dynamic and pleiomorphic, and their size varies depending on the cell type and growth conditions. Vacuoles compartmentalize different cellular components such as proteins, sugars, ions and other secondary metabolites and play critical roles in plants response to different biotic/abiotic signaling pathways. In this review, we will summarize the patterns of changes in vacuole morphology in certain cell types, our understanding of the mechanisms of plant vacuole biogenesis, and the role of SNAREs and Rab GTPases in vacuolar trafficking.

## INTRODUCTION

Plant vacuoles are large compartments that occupy a significant volume (up to 90%) of plant cells. Under normal growth conditions water can flow into the vacuole, building up the turgor pressure that drives cell wall expansion. Under osmotic stress conditions, water flows out of vacuoles and the turgor from the vacuole is reduced; thus plant cell growth is impaired. The simple physical phenomenon of turgor pressure driving cell expansion is under the control of multiple complex biological processes. One of these is the response of vacuoles to different growth conditions and cellular signaling to control cell growth. This makes it very important to study the regulation of vacuole morphology and trafficking pathways to the vacuole. Vacuoles are also the storage organelles for proteins, sugars, and other metabolites and these storage vacuoles may exist in the same cell as the lytic vacuoles (Paris et al., 1996; Olbrich et al., 2007). On the topic of protein trafficking to the storage vacuoles, the readers are referred to excellent reviews (Robinson et al., 2005; Vitale and Hinz, 2005; Xiang et al., 2012; Robinson, 2014). In this article, we will discuss our understanding of the dynamic changes of vacuole morphology, the mechanisms of vacuole biogenesis during plant development, and the advantage of chemical biology in studying this process.

## VACUOLES CHANGE MORPHOLOGY DURING GROWTH AND IN RESPONSE TO ENVIRONMENT

Plant vacuoles have different morphology and dynamics in different cell types or in the same cell under different conditions. The development of GFP-tagged vacuolar markers makes it possible to study the dynamic changes of vacuole morphology under different growth conditions as well as to study the cellular components that affect vacuole morphology (Cutler et al., 2000; Uemura et al., 2002; Reisen et al., 2005). Vacuole morphology in stomata guard cells, cultured tobacco cells, and *Arabidopsis* pavement cells under different conditions has been documented to study the relationships between vacuole morphology and plant response to biotic and abiotic signaling.

Plant stomata aperture is regulated by hormones, light, and water flow (Araujo et al., 2011). Stomatal closure or opening is accompanied by changes in vacuole morphology in guard cells and is an excellent system for studying the relationship between vacuole biogenesis and cell shape control (Gao et al., 2005, 2009; Tanaka et al., 2007; Bak et al., 2013). Gao et al. (2005) studied the dynamic changes of vacuole morphology during stomatal movement using fluorescent dyes that labeled vacuoles in *Vicia faba* guard cells. When the stomata were closed, the guard cells contained a large number of small spherical vacuoles with a diameter of 1–5 μm. When stomata start to open, the number of vacuoles decreases and the size of vacuoles increase. Observations from single confocal image slices found that the small vacuoles fuse with each other to form larger vacuoles during stomatal opening. Subsequently, the large vacuoles split into many small vacuoles during stomata closing. There are also complex vacuolar structures such as tubular vacuolar membranes in partially open stomata guard cells, invaginations of the tonoplast, and vesicle structures inside the vacuole lumen. The same pattern of change in vacuole morphology during stomatal movement was observed in *Arabidopsis* guard cells expressing the GFP-AtVAM3 vacuole marker (Tanaka et al., 2007). However, these seemed small individual vacuolar compartments actually belong to the same continuous tonoplast membrane and form interconnected lumen, supported by 3-D image reconstruction and photobleaching experiments (Gao et al., 2005; Tanaka et al., 2007). This indicates that during stomata closing, vacuole membranes form convoluted structures rather than undergo complete fission to form individual smaller vacuole compartments. However, during stomata opening, the vacuole membrane becomes less convoluted probably due to the change of vacuole lumen content.

Another cell type that has been used to study the relationship between changes in vacuole morphology and cell shape control is the *Arabidopsis* epidermal pavement cell. *Arabidopsis* cotyledon epidermal pavement cells experience alternated phases of lobe initiation and lateral isotropic cell expansion to build the complex jigsaw puzzle piece shape (Zhang et al., 2011). Hawes et al. (2001) used γ-TIP-GFP to label the lytic vacuole in leaf epidermal cells of *Arabidopsis* to study vacuole biogenesis during pavement cell development. They found that γ-TIP-GFP not only labeled the main vacuole whose boundary was appressed to the plasma membrane but also labeled transvacuolar strands and spherical vacuoles/vesicles within the main vacuole. These spherical vacuolar structures move within the major vacuole and change shape frequently but do not fuse with each other. Also using γ-TIP-GFP, Saito et al. (2002) studied vacuole morphology in different stages of *Arabidopsis* cotyledon pavement cells, and they found similar spherical membrane structures, which they called “bulbs”, within the vacuole lumen. The frequency of these bulbs decreases with the progression of pavement cell development and their frequency is reduced under starvation. Transmission electron microscopy imaging and 3-D reconstruction showed that these intricately folded bulbs are not due to fluorescence tag expression but are continuous with the main vacuole membrane (Saito et al., 2002). Uemura et al. (2002) also found similar vacuole morphology in *Arabidopsis* leaf epidermal cells using GFP-AtVAM3 as a marker. Three-dimensional reconstruction and time-lapse imaging reveal the continuity and dynamic nature of vacuolar membrane as well. It has been proposed that the bulbs are vacuolar invaginations leading to protein degradation (Maitrejean and Vitale, 2011). However, a very recent report shows that the bulbs observed from GFP-tagged tonoplast proteins are due to the dimerization of GFP protein (Segami et al., 2014). The authors show that the frequency of bulbs labeled by GFP-VHP1 (vacuolar H^+^-pyrophosphatase) was significantly reduced when a non-dimerizing GFP (mGFP) was used as the tag. Instead, some intravacuolar spherical structures different than bulbs were observed in mGFP-VHP1 line and these spherical structures seemed also in continuity with the vacuole membrane. Thus, although it seems that multiple vacuoles with different sizes and shapes exist in the same cell, they are generally from the same continuous membrane. How does the same membrane become convoluted and form complicated shape is not well understood. Another point worth mentioning is that the normally observed transvacuolar strands are actually in a sheet-like structure and show another aspect of the complexity of vacuole morphology (Uemura et al., 2002).

Cultured tobacco cells are also excellent to study the control of vacuole morphology. Similar to the situation in stomata guard cells and in the *Arabidopsis* epidermal cells, single slice confocal images in tobacco-cultured cells seem to indicate the existence of several individual vacuoles, but 3-D reconstruction again indicates a convoluted continuous tonoplast (Mitsuhashi et al., 2000; Reisen et al., 2005). Very dynamic transvacuolar strands with different shapes extend from the nuclear region to the cell periphery and radiate throughout the vacuole in these cultured cells (Reisen et al., 2005; Ruthardt et al., 2005). Vacuoles in pollen tubes are also very dynamic and have complex tubular structures and invaginations, and it is tempting to speculate that these complex structures might also belong to the same membrane (Hicks et al., 2004).

Vacuoles are responsive to plant environmental growth conditions. Changes in vacuole morphology due to water stress were documented a long time ago using electron microscopy in maize and sorghum leaf mesophyll cells (Giles et al., 1974, 1976). When the water potential in maize leaf mesophyll cells reached –18.5 bars, the tonoplast broke down in parallel with the cell disruption (Giles et al., 1974). However, in sorghum leaf mesophyll cells, even when water potential reached –37 bars, the tonoplast did not break and smaller vacuoles were formed instead, which might due to vacuole fragmentation (Giles et al., 1976). When water stress ceases, vacuoles return to their normal morphology quickly. The small vacuoles observed by Giles et al. in sorghum leaf mesophyll cells could belong to the same convoluted vacuole membrane. Osmotic stress also induces vacuole membrane convolution in cultured tobacco cells (Reisen et al., 2005). The difference between maize and sorghum vacuoles in response to water stress indicates regulatory machinery for vacuole membrane convolution that could contribute to plant survival under these unfavorable growth conditions. This notion is supported by the fact that altering expression levels of genes involved in vacuole morphology regulation affected plant sensitivity to water or other abiotic stresses (Mazel et al., 2004; Leshem et al., 2010). These evidences reveal important roles of vacuole morphology in plant response to environment besides correlating with plant growth and development.

## CELLULAR MECHANISMS THAT CONTROL VACUOLE MORPHOLOGY

Vacuoles are part of the secretory pathway that originates from the endoplasmic reticulum (ER) where lipids and secreted proteins are synthesized. One route for vacuole transport involves the conventional vesicle trafficking pathway that requires COPII vesicle-mediated ER export and subsequent Golgi and post-Golgi transport. This trafficking pathway to the vacuole has been recently reviewed (Xiang et al., 2012; Viotti, 2014). Another pathway to the vacuole involves direct transport from the ER to the vacuole that is independent of COPII vesicle-mediated ER export via Golgi and post-Golgi vesicles. The existence of direct trafficking from ER to the vacuole is supported by the facts that membrane cargos can be transported to the vacuole even when Golgi and post-Golgi trafficking was blocked by either chemical treatments or dominant-negative GTPase overexpression (Gomez and Chrispeels, 1993; Batistic et al., 2009; Bottanelli et al., 2011b; De Marchis et al., 2013; Viotti et al., 2013). There is additional evidence showing that autophagy is involved in direct transport of storage proteins from the ER to the vacuole (Herman and Schmidt, 2004; Reyes et al., 2011; Honig et al., 2012). It was proposed that direct trafficking of storage proteins from ER to vacuole involves direct budding of vesicles containing vacuole cargos, the engulfment of these vesicles by the phagophore membrane, and the fusion of autophagosomes with the tonoplast (Michaeli et al., 2014). However, Viotti et al. (2013) reported the observation of double layered provacuoles whose biogenesis is independent of either Golgi or autophagy machinery. In fact, they found rare incidences of a direct connection between ER and provacuoles using electron microscopy. This supports the notion that provacuoles originate directly from subdomains of the ER and may fuse to the preexisting vacuole network using the homotypic vacuole fusion machinery. Different models of ER to vacuole trafficking, whether through autophagy or direct fusion, may result from the difference in types of cargos, stages of vacuole biogenesis, and tissues or experimental conditions. It is possible that one pathway is dominant in certain circumstances. For example, under ER stress conditions, it is convincing that ER lumen proteins are transported to the vacuole for degradation through authophagy by a mechanism that bypasses Golgi and post-Golgi prevacuolar compartments (Liu et al., 2012).

Membrane proteins destined to the vacuole for degradation accumulate in prevacuolar compartments or late endosomes and fuse with preexisting vacuoles (Scheuring et al., 2011). The homotypic membrane fusion step is essential in vacuole biogenesis and in some cases different types of vacuoles such as protein storage vacuoles and lytic vacuoles can also fuse to generate a large central vacuole (Zheng and Staehelin, 2011). The exact components required for vacuole fusion in plants are not known because there is no *in vitro* vacuole fusion assay that has been developed. Understanding plant vacuole fusion mechanism is assisted by the conservation between the plant and yeast vacuole fusion machinery. The mechanisms of vacuole fusion are well studied in yeast because of straightforward genetic screens for mutants with defects in vacuole morphology and the development of excellent *in vitro* vacuole fusion assays (Wada et al., 1992; Haas et al., 1994; Seeley et al., 2002; Merz and Wickner, 2004; Jun and Wickner, 2007). A successful vacuole fusion event involves tethering, docking and fusion processes which require Rab-family GTPase (Ypt7p/Vam4p), vacuolar SNAREs (Nyv1p, Vti1p, Vam3p and Vam7p), homotypic fusion and vacuole protein sorting (HOPS) complex (Vps11p, Vps16p, Vps18p, Vps33p, Vps39p and Vps41p), and certain lipids, as has been reviewed in detail (Armstrong, 2010; Wickner, 2010). Each of these vacuole fusion components exists in the *Arabidopsis* genome and some of the homologs have been studied and proven to have function in plant vacuole fusion. We will discuss the role of these components in plant vacuole biogenesis in the rest of this section. The names, cellular localization and loss of function phenotypes of *Arabidopsis* proteins involved in the vacuole fusion process are listed in **Table 1**.

**Table 1:** Names, cellular localization, and functions of proteins involved in vacuole fusion.

*Arabidopsis* protein name	Yeast homolog	Cellular localization	Function	Loss of function phenotypes
AtRabG3c	Ypt7p/Vam4p	Tonoplast	Activate HOPS complex	Inhibits vacuole targeting of both soluble and membrane proteins
AtRabG3f	Vpt7p/Vam4p	Prevacuolar compartments and the tonoplast	Activate HOPS complex	Seedling lethality, has enlarged prevacuolar compartments and produces smaller sized vacuoles, inhibits protein trafficking to the vacuole
AtVAM3/SYP22	vam3p	Predominantly in vacuoles	t-SNAR	Pleiotropic growth phenotypes including dwarf plant and wavy leaves
AtPEP12/SYP21	vam3p	Predominantly in prevacuolar compartments	t-SNARE	Function redundantly with AtVAM3/SYP22
VTI11	Vti1p	Prevacuolar compartment	v-SNARE	Affect trafficking of lytic vacuole cargos and homotypic vacuole fusion
VTI12	Vti1p	TGN	v-SNARE	Affects trafficking of storage proteins to the vacuole
VCL1 (vacuoless1)	Vps16p	Vacuole and prevacuolar compartments	A component of HOPS complex	Null mutants do not have lytic vacuoles

Like in yeast, *Arabidopsis* Rab GTPases are involved in vacuole fusion. The *Arabidopsis* Rab GTPases G subfamily (AtRabG) has significant similarity to human Rab7 and yeast Ypt7p/Vam4p RabGTPases and contains eight members (Vernoud et al., 2003; Woollard and Moore, 2008). The relatively large size of the family makes it difficult to study the contribution of each member to the vacuole fusion process using genetic knockout approaches. As in the case of other small GTPases, overexpression of dominant-negative forms of Rab GTPases has been used to study loss-of-function phenotypes. For example, RabG3c is localized to the tonoplast and dominant-negative RabG3c inhibits vacuolar targeting of both soluble and membrane proteins (Bottanelli et al., 2011a,b). RabG3f was found to localize to both the prevacuolar compartment and the tonoplast. Dominant-negative RabG3f overexpression causes enlarged prevacuolar compartments, alters vacuole morphology by producing smaller sized fragmented vacuoles, and inhibits protein vacuolar trafficking (Cui et al., 2014). RabG3f is activated by the MON1/SAND-CCZ1 complex, which is an effecter of Rab5 GTPase (Cui et al., 2014; Singh et al., 2014). Mutants of *mon1/sand* and *ccz1* have defects in vacuole storage protein trafficking and their prevacuolar compartments fail to fuse normally (Cui et al., 2014; Ebine et al., 2014; Singh et al., 2014). Genetic studies by Singh et al. using different vacuolar cargos revealed that plants not only use the sequential action of Rab5 (*Arabidopsis* Rab F family) and Rab7 (*Arabidopsis* Rab G family) in regulating vacuole trafficking, but also contain Rab5- and Rab7-independent vacuolar trafficking pathways (Singh et al., 2014).

SNARE proteins play an important role in vesicle fusion and also exist as a larger family in *Arabidopsis* compared with yeast (Sanderfoot et al., 2000). Some SNARE proteins involved in vacuole fusion have been characterized at the genetic and cellular levels. The *Arabidopsis* t-SNARE gene AtVAM3/SYP22 can complement the vacuole morphology phenotype of a yeast *vam3p* deletion mutant, and it is localized to the vacuoles and prevacuolar compartments (Sato et al., 1997; Sanderfoot et al., 1999). AtVAM3/SYP22 functions interchangeably with its close homolog AtPEP12/SYP21 in plant development, and they both can incorporate into a complex with SYP51 and VTI11 (Sanderfoot et al., 2001; Uemura et al., 2010). There are three *Arabidopsis* SNAREs that are similar to yeast Vti1p and at least two can substitute for different aspects of yeast Vti1 function (Zheng et al., 1999; Sanderfoot et al., 2000). VTI11 and VTI12 can substitute for each other in SNARE complexes where they function redundantly and interchangeably in plant growth regulation (Sanderfoot et al., 2001; Surpin et al., 2003); however, at the cellular level they play specialized roles in trafficking to storage and lytic vacuoles (Sanmartin et al., 2007). VTI11 affects the trafficking of lytic vacuolar cargos and VTI12 affects the trafficking of storage vacuolar cargos. A SNARE complex containing SYP61/SYP41/VTI12 is regulated by TGN-localized AtVPS45 protein and is involved in retrograde transport of vacuolar sorting receptors back to the TGN (Zouhar et al., 2009). Embryo lethality in the *vit11*;*vit12* double mutant indicates that the pathway regulated by these SNAREs is essential for plant development (Surpin et al., 2003). A recently identified weak allele of *VTI11* shows directly that VTI11 is involved in the homotypic fusion of both storage and lytic vacuoles (Zheng et al., 2014). The dynamic cycle of SNARE associations with other components during plant vacuole fusion is not well understood.

Homologs of the HOPS complex components are single genes in *Arabidopsis* and are essential for plant development because knockout mutants are embryonic lethal (Rojo et al., 2001; Niihama et al., 2009). *Arabidopsis* VACUOLESS1 (VCL1) encodes yeast Vps16p homolog, which is one of the HOPS complex proteins (Seals et al., 2000; Wurmser et al., 2000; Rojo et al., 2001). VCL1 together with other two HOPS proteins AtVPS11 and AtVPS33 are localized to the tonoplast and prevacuolar compartments, which is consistent with their roles in vacuole fusion (Rojo et al., 2003). Embryos of *vcl1* do not contain lytic vacuoles and have autophagosome accumulation instead, which confirms the role of HOPS complex proteins in vacuole fusion in plants (Rojo et al., 2001). Further study on plant HOPS complex assembly and regulation will help understanding the mechanisms of plant vacuole fusion.

Besides proteins, special lipids are also required for proper vacuole fusion (Seeley et al., 2002; Wickner, 2010). In yeast, vacuole fusion requires phosphatidylinositol-3-phosphate [PI(3)P], phosphatidylinositol-4,5-biphosphate [PI(4,5)P2], diacylglycerol (DAG) and the yeast sterol ERG (Mayer et al., 2000; Kato and Wickner, 2001; Seeley et al., 2002; Fratti et al., 2004). *In vitro* vacuole fusion assays in yeast show that membrane fusion proteins and regulatory lipids are assembled to the vertex ring in an interdependent manner rather than either proteins or lipids alone (Fratti et al., 2004). In plants, we do not know the exact lipids required for the fusion process, but there is evidence for the involvement of certain lipids in vacuole fusion at genetic level. Novakova et al. (2014) recently characterized a group of tonoplast localized suppressor of actin (SAC) family phosphoinositide phosphatase genes and found the involvement of phosphoinositides in vacuole morphology. Loss-of-function *sac* mutants have altered levels of phosphoinositides and also have defects in the fusion of both lytic and storage vacuoles. *Arabidopsis* PI(3)P 5-kinase (PI3P5K) mutants (combinations of *fab1a* and *fab1b*) have severe defects in pollen vacuole morphology, indicating the role of phosphatidylinositol-3,5-biphosphate [PI(3,5)P2] in vacuole biogenesis (Whitley et al., 2009). In fava bean (*V. faba*), an inhibitor of PI3P5K delays changes in vacuole morphology in guard cells during abscisic acid (ABA)-induced stomatal closure which also indicates the role of PI(3,5)P2 in vacuole fusion (Bak et al., 2013).

Although the required components for vacuole morphology are conserved between plants and yeast, it is apparent that plant vacuole morphology is more complicated and is essential to plant development. Further investigation of the dynamic interaction network between these components will reveal more details about plant vacuole biogenesis process.

## OTHER FACTORS THAT REGULATE VACUOLE MORPHOLOGY

Besides the components of vacuole biogenesis machinery, there are other factors affecting vacuole morphology. Screens for vacuole morphology defects in non-essential yeast deletion mutants not only identified genes involved in catalyzing direct vacuole fusion but also genes in lipid metabolism, GTPase effectors, protein kinases, and cytoskeletal proteins among others (Seeley et al., 2002). Many of these non-essential genes interact with essential genes involved in vacuole fusion and are part of the signaling cascade regulating vacuole morphology. In plants, point mutation lines carrying a fluorescent vacuole marker have been screened with confocal microscopy for vacuole biogenesis defects (Avila et al., 2003). Of 9175 seedlings that were screened, 211 had defects in vacuole biogenesis. One hundred and ten of these produced seeds in the next generation. The relatively high frequency of vacuole morphology defects indicates that there are multiple factors regulating vacuole morphology. The phenotypes in these mutants include increased numbers of small vesicles, large aggregates of the fluorescence marker GFP:δTIP, hyper-dynamic transvacuolar strands and complex vacuoles. High percentage of infertile seedlings in mutants carrying the complex vacuole phenotypes was a result of the essential role of plant vacuoles in development and this limits our understanding of plant vacuole biogenesis pathways using these mutants (Rojo et al., 2001; Avila et al., 2003). In comparison with yeast, it takes longer time to clone causal genes in plants and this also slows down our understanding of the signaling cascade components involved in vacuole biogenesis. One of the 110 sterile lines was further characterized and revealed that a myrosinase-associated protein might regulate early trafficking pathways. The underlying mechanism is not clear but points to our need for further investigation (Agee et al., 2009).

Actin cytoskeleton is involved in vacuole morphology in both yeast and plants. Actin is detected in isolated vacuoles of both yeast and plant cells (Eitzen et al., 2002; Mathur et al., 2003; Carter et al., 2004; Higaki et al., 2006; Sheahan et al., 2007). In yeast, actin is enriched at the vacuolar membrane domain where fusion occurs. Introducing point mutations in actin or deletions of actin motor or nucleator genes causes vacuole fusion defects (Eitzen et al., 2002). Plant actin bundles associate with transvacuolar strands and function together with myosin motor proteins to promote the formation, rearrangement and rapid movement of transvacuolar strands (Hoffmann and Nebenfuhr, 2004; Higaki et al., 2006; Sheahan et al., 2007; Szymanski and Cosgrove, 2009). Upon microinjection of profilin, which disrupts actin cytoskeleton in living plant cells, transvacuolar strands quickly collapse. This is one of the earliest evidences indicating the critical role of actin cytoskeleton in maintaining plant vacuolar structure and dynamics (Staiger et al., 1994). Drug disruption of actin organization in *Arabidopsis* guard cells results in inhibition of vacuole fusion during stomatal opening (Li et al., 2012). However it is not clear at a mechanistic level how actin affects transvacuolar strand formation and vacuole fusion. Some evidences show that the evolutionary conserved actin nucleator actin-related protein 2/3 (ARP2/3) complex is involved in the regulation of vacuole fusion. There are vacuole fusion defects in trichomes and guard cells of some arp2/3 subunit mutants (Mathur et al., 2003; Li et al., 2012). In *Arabidopsis*, the ARP2/3 complex has a different localization pattern than its activator, the five-subunit WAVE/SCAR complex (Yanagisawa et al., 2013; Zhang et al., 2013a,c). It is unclear how the ER-localized WAVE/SCAR actin nucleation activation signal is transmitted to a different cellular compartment to influence the vacuole fusion process. The relatively weak growth phenotypes of mutants in both WAVE/SCAR and ARP2/3 complex genes and the relatively minor vacuole fusion phenotypes displayed in some of these mutants indicates that there are other actin regulatory pathways involved in transvacuolar strand formation and vacuole fusion.

Rho GTPase signaling pathways are upstream regulators of actin organization and are involved in vacuole fusion in yeast (Eitzen et al., 2001; Muller et al., 2001; Seeley et al., 2002; Jones et al., 2009; Logan et al., 2010; Estravis et al., 2011). Cdc42p regulates vacuole membrane fusion through regulation of actin polymerization (Isgandarova et al., 2007). Vacuoles from a cdc42 temperature-sensitive mutant have vacuole fusion defects when grown at restrictive temperature and a dominant negative cdc42 mutant has vacuole fusion defects under hypotonic stress induction (Muller et al., 2001; Jones et al., 2009). Both Rho1p and Cdc42p have a direct role in mediating the docking stage of homotypic vacuole fusion downstream of Rab GTPase Ypt7p tethering but upstream of trans-SNARE priming (Eitzen et al., 2001; Muller et al., 2001). Like yeast Rho GTPases that are enriched in vacuole membrane extracts (Muller et al., 2001), Rho GTPases of Plants (ROPs) are localized to the tonoplast in pea tapetal cells (Lin et al., 2001). Although there is no direct evidence showing the effect ROPs in vacuole morphology at the genetic level, ROPs have been shown to regulate both cytoskeleton organization and vesicle trafficking. This indicates that ROP signaling may affect vacuole morphology in plants as well (Qiu et al., 2002; Basu et al., 2008; Craddock et al., 2012; Zhang et al., 2013). The *Arabidopsis* DOCK family ROP guanine nucleotide exchange factor (GEF) protein SPIKE1 is localized to ER exit sites (ERES) and participates in the maintenance of ER homeostasis by promoting the formation of ERES (Zhang et al., 2013b). Although it is not clear what cargo proteins are affected by SPIKE1 in the early secretory pathway, it will not be surprising if SPIKE1 affects the trafficking of proteins that control vacuole morphology. It is also known that SPIKE1 associates with the ER-localized WAVE/SCAR complex to incorporate the ROP signaling pathway into the regulation of actin organization and thus may affect vacuole morphology (Basu et al., 2008). We would gain insights into the role of ROP GTPases in vacuole biogenesis by studying the mechanisms of temporal–spatial regulation of ROP signaling on trafficking and cytoskeleton organization.

Because vacuoles are positioned at the end of the secretory pathway, mutations in genes involved in upstream trafficking can result in vacuole morphology defects. For example, the TGN/EE (early endosome)-localized protein KEG affects plant vacuole morphology and transport to the vacuole (Gu and Innes, 2012). There is enhanced vacuole fragmentation in different cell types in the *keg* mutant and brassinosteroid receptor BRI1 trafficking to the vacuole is significantly inhibited. The linkage between KEG and vacuole morphology is not clear. Microscopy based forward genetic screening for proteins that affect trafficking using GFP-tagged PIN1 as a marker revealed the role of adaptor protein complex-3 (AP-3) in vacuole morphology. Loss-of-function mutants of *ap-3*β, *AP-3*δ, and *ap-3*μ*^DN^* have similar vacuole morphology defects (Feraru et al., 2010; Zwiewka et al., 2011). As these mutants also cause intracellular accumulation of proteins such as PIN1 and aleurain in structures distinct from BFA bodies, it is likely that the AP-3 complex is involved in upstream trafficking pathways (Feraru et al., 2010). Multiple layers of mature vacuoles were observed in mutants with disrupted AP-3β and VPS45 function, both of which are upstream regulators. This indicates that the post-Golgi trafficking machinery is required for downstream vacuole formation (Viotti et al., 2013).

## CONTRIBUTION OF CHEMICAL BIOLOGY TOWARD UNDERSTANDING VACUOLE MORPHOLOGY AND TRAFFICKING

As discussed above, plant vacuoles are essential for growth and development and interact with other components of the vesicle trafficking system. There is either genetic redundancy or mutant lethality in many critical genes involved in the vacuolar trafficking machinery. Also, the phenotypes we observe in mutants reflect the consequence of equilibrated cellular processes and thus can be misleading. For example, mutations in an upstream trafficking pathway may cause a vacuole morphology phenotype. Chemical biology uses small molecules to disrupt cellular processes and has the advantage of being transient, reversible and dosage dependable (Hicks and Raikhel, 2010, 2012). There are good examples for the application of chemical biology in studying vacuolar trafficking.

Based on the conservation between plant and yeast vacuolar trafficking machineries, Zouhar et al. (2004) screened a library for chemicals that inhibited trafficking of vacuole-targeted carboxypeptidase Y (CPY) in yeast. Further characterization of thirteen compounds that were active in yeast identified three compounds named sortins that disrupted protein vacuolar trafficking and vacuole morphology in *Arabidopsis* (Zouhar et al., 2004). Genetic screening and characterization of mutants that were hypersensitive to sortin1 showed that a properly regulated flavonoid metabolism pathway participated in regulating vacuole morphology (Rosado et al., 2011).

Chemical library screening for compounds that disrupt plant endomembrane trafficking using tobacco pollen as a primary screen, combined with secondary screening using *Arabidopsis* seedlings expressing cellular markers that are cycled within the secretory pathway, identified 360 compounds that inhibited plant endomembrane trafficking (Drakakaki et al., 2011). Some of these 360 compounds affect trafficking to the vacuole. For example, trafficking of PIN auxin transporters to the vacuole is enhanced upon ES5 treatment (Drakakaki et al., 2011). Further screening of these 360 compounds using *Arabidopsis* seedlings expressing GFP-TIP2:1 and mCherry-HDEL identified compounds that cause tonoplast localized TIP2:1 to accumulate in the ER or intermediate membrane compartments (Rivera-Serrano et al., 2012). One interesting compound from this further screen is C834, which affects α-TIP and δ-TIP but not γ-TIP indicating that it can differentiate two vacuole trafficking pathways. Further genetic screening for altered sensitivities to these chemicals will identify cognate targets and contribute to our understanding of the mechanisms of vacuole morphology and trafficking.

Synthetic ceramide PDMP (D-L-threo-1-phenyl-2-decanoyl amino-3-morpholino-1-propanol) competes with natural ceramide and inhibits sphingolipid biosynthesis (Vunnam and Radin, 1980; Asano, 2003). It was recently found that PDMP induced vacuole membrane invaginations that contain cytoplasmic contents in *Arabidopsis* upon short-term treatment (hours) at low dosage (Kruger et al., 2012). It is not clear how the PDMP-targeted sphingolipid metabolism pathway affects plant vacuole morphology.

## CONCLUSION

Vacuoles have complex morphology in different cell types and at different stages of plant development. Vacuole morphology is affected by environmental conditions such as water and nutrition. Located at the distal end of the endomembrane trafficking pathway, vacuoles receive signals from both anterograde transport from the ER and recycled membrane and proteins from the plasma membrane. The studies in yeast give hints about the mechanisms of plant vacuole morphology and trafficking, but it is clear that plants have evolved specific vacuolar trafficking regulatory pathways. An *in vitro* vacuole fusion system has not been reported in plant cells, partly due to the complexity of the plant vacuole biogenesis machinery. Although some components that are required for vacuole fusion have been discovered, the sequential interactions between these components are not well characterized. The regulatory network that involves these components also needs to be studied. At the same time, it is meaningful to study the dynamic regulation of vacuolar trafficking in the context of the entire cellular system. For example, in certain cell types at specific developmental stages, how do cells balance the quantity and flux of protein and lipids that are delivered to the vacuole versus that delivered/recycled to the plasma membrane? In some cell types that have storage vacuoles, this question is of great importance. Careful live cell imaging and quantification using selected cargoes could help answering this question.

Genetic redundancy or mutant lethality in essential vacuole biogenesis and trafficking genes limit our understanding the mechanisms of vacuole biogenesis using knockout mutants. The dynamic nature of the endomembrane trafficking system also limits the value of constitutive loss-of-function mutants. Chemical biology presents great advantages in studying dynamic cellular processes such as vacuole biogenesis. Screening for chemicals that target specific vacuolar biogenesis steps or target specific proteins allows transient blocking of specific steps and the instant cellular response can be observed. This is of great value to avoid artifacts from feedback response. Chemical treatment combined with conditional genetic screens, such as chemical hypersensitive and resistant screens, would allow the identification of new components related to vacuole biogenesis. Some growth-related phenotypes induced by chemical treatment could be used in this kind of screening, such as root elongation or gravitropic response. However, a more direct way is to combine chemical screening with high-throughput cellular imaging to identify mutant genes that alter transient responses to the chemical at the cellular level. In this manner, new components involved in pathways of interest can be discovered. In the past, chemical libraries have been screened to identify small molecules that interrupt plant endomembrane trafficking. Subsequent chemical characterization and target identification can be a great challenge. With the tools of mass spectrometry and nuclear magnetic resonance spectrometry, it is possible to screen chemicals that directly target proteins of interest. This is more helpful for studying particular cellular processes of interest.

It remains largely unknown how vacuole-localized proteins and vacuolar membrane lipids interact with cytoskeleton and signaling pathways to affect vacuole biogenesis. Many proteins that reside at the vacuole membrane are not well characterized. The network of interactions between vacuole proteins and cytoskeleton regulators is not well established. A systematic characterization of protein–protein and protein–lipid interactions will provide critical information on this topic. Live cell imaging of fluorescently tagged vacuole membrane and cytoskeleton regulator markers using newly developed super-resolution imaging and light-sheet microscopy would allow us to study the dynamic correlation between these proteins and provide real time evidence of modes of action.

## Conflict of Interest Statement

The authors declare that the research was conducted in the absence of any commercial or financial relationships that could be construed as a potential conflict of interest.
